# The roles of soybean lecithin in aquafeed: a crucial need and update

**DOI:** 10.3389/fvets.2023.1188659

**Published:** 2023-09-15

**Authors:** Wendy Wee, Guillermo Téllez-Isaías, Zulhisyam Abdul Kari, Romalee Cheadoloh, Muhammad Anamul Kabir, Khairiyah Mat, Suniza Anis Mohamad Sukri, Mohammad Mijanur Rahman, Nor Dini Rusli, Lee Seong Wei

**Affiliations:** ^1^Center of Fundamental and Continuing Education, Universiti Malaysia Terengganu, Terengganu, Malaysia; ^2^Department of Poultry Science, University of Arkansas, Fayetteville, AR, United States; ^3^Department of Agricultural Sciences, Faculty of Agro-Based Industry, Universiti Malaysia Kelantan, Jeli Campus, Kelantan, Malaysia; ^4^Advanced Livestock and Aquaculture Research Group, Faculty of Agro-Based Industry, Universiti Malaysia Kelantan, Jeli Campus, Kelantan, Malaysia; ^5^Faculty of Science Technology and Agriculture, Yala Rajabhat University, Yala, Thailand; ^6^Department of Aquaculture, Sylhet Agricultural University, Sylhet, Bangladesh

**Keywords:** lecithin, growth performance, feed utilization, immunity, antioxidant, stressor, sustainable aquaculture

## Abstract

Soybean lecithin is extensively used as the dietary supplementation of phospholipids in animal production. Soybean lecithin plays significant roles in aquafeed as growth promoter, feed enhancer, immunity modulator and antioxidant activity stimulator for aquaculture species. Besides, soybean lecithin is also reported to help aquaculture species being resilient to physical and chemical stressors. In this review, common sources, chemical structure and mode of action of lecithin, with highlight on soybean lecithin application in aquaculture over four-decadal studies published between 1983 and 2023, were evaluated and summarized. By far, soybean lecithin is best-known for its beneficial effects, availability yet cost-effective for aquafeed formulation. Findings from this review also demonstrate that although nutritional profile of long-chain polyunsaturated fatty acids and phosphatidylcholine from egg yolk and marine sources are superior to those from plant sources such as soybean, it is rather costly for sustainable application in aquafeed formulation. Moreover, commercially available products that incorporate soybean lecithin with other feed additives are promising to boost aquaculture production. Overall, effects of soybean lecithin supplementation are well-recognized on larval and juvenile of aquaculture species which having limited ability to biosynthesis phospholipids *de novo*, and correspondingly attribute to phospholipid, a primary component of soybean lecithin, that is essential for rapid growth during early stages development. In addition, soybean lecithin supplementation plays a distinguish role in stimulating maturation of gonadal development in the adults, especially for crustaceans.

## 1. Introduction

Aquafeed is the main production expense of an aquaculture operation, accounting for about 50 to 70% of the total aquaculture operation cost. According to a recent study, aquafeed costs about 65% of the total aquaculture production cost (1). The feed cost is expected to further increase due to excessive reliance on conventional raw materials, especially fish oil and fish meal in aquafeed formulation (2). Sustainability of the aquaculture industry can be greatly impacted by the future shortages of fish oil and fish meal (3). Hence, it is vital to have some animal origin materials gradually replaced by substances derived from plant origin such as soybean lecithin, soybean oil, and soybean meal in the aquafeed formulation.

As a byproduct of the oilseed industry, lecithin is widely introduced into food, cosmetic, pharmaceutical and other non-food industries as the emulsifier and liposomes producer, along with their great nutritional value (4). In the aquaculture industry, lecithin becomes one of the important raw materials to meet essential fatty acid requirements of the targeted species (5). Lecithin production became well-established in the United States in 1940 and expanded rapidly upon the commercial introduction of genetically modified (GMO) soybeans in 1996. On the other hand, lecithin from non–GMO sources like sunflower, rapeseed, and rice bran are favored by the European market (6, 7). Soybean lecithin is currently the primary source available in the worldwide market and offers a comparatively affordable price compared to lecithin from other sources, especially the marine lecithin (8). Commercial soybean lecithin claimed to consist high concentration of phospholipid, 65–75% (9) while some other studies showed that concentration of phospholipids in soybean lecithin ranged between 28–44% (10), 55–57% (11), 47% (12), and 38–45% (13). These differences were probably due to the discrepancies in the purity of soybean lecithin and approaches used to determine the concentration of phospholipid in soybean lecithin.

Aquafeed formulation that offer phospholipid composition resembles to the fish egg is deemed to be an ideal diet for fish larvae to ensure maintenance and functionality of cellular membrane structure, and as a source of energy to the fish (14). Therefore, phospholipid is supplemented in the larval diet of the aquatic animals owing to their limited biosynthetic capacity in the initial stages (15–17). For most of the aquaculture species, a supplementation of 8 to 12% dietary phospholipid is regarded to be optimal to promote growth and survival (18). Nonetheless, requirements for dietary phospholipids vary among species, life stage of the species, and the source and purity of the phospholipids. Phospholipid from different sources of lecithin such as soybean, milk, egg and krill, differs from one source to another in terms of phospholipid classes and fatty acids nature (19). Remarkably, soybean lecithin predominated by phosphatidylcholine, followed by phosphatidylethanolamine, and phosphatidylinositol provides an excellent source of phospholipids and fatty acids which are essential to aquatic animals during their early life stages (20). In this review, common sources, chemical structure and mode of action of lecithin are presented with the examples on how soybean lecithin promotes aquaculture production by improving feed utilization, growth performance, intestinal health, antioxidant capacity and resilience against stressors.

## 2. Lecithin sources and chemical structure

Literally means egg yolk in Greek word *lekithos*, lecithin was first extracted from the egg yolk by a French chemist, Theodore Nicolas Gobley in 1850. Subsequently, soybean lecithin came into worldwide commercial availability in 1921, almost a decade after the introduction of soybean from China into Europe and North America (21, 22). Global lecithin market size has exceeded USD 2 billion in 2021 and is forecasted to surpass USD 3.4 billion by 2030 (23). Lecithin is a complex mixture of phosphatide fraction available in both plants and animals, especially in the soybean and egg yolk (19). As a result of the esterification of choline, ethanolamine, serine, and inositol to a phosphatidic acid backbone, phospholipids are formed and classified into respective phosphatidylcholine (PC), phosphatidylethanolamine (PE), phosphatidylserine (PS) and phosphatidylinositol (PI) with other constituents of fatty acids, triglycerides, and carbohydrates (4). Despite that the term for lecithin specifically refers to phosphatidylcholines in some earlier literatures, it is often used interchangeably with phospholipids since the commercial lecithin is largely made up of phospholipids and vegetable oils (24, 25).

**Figure 1:**
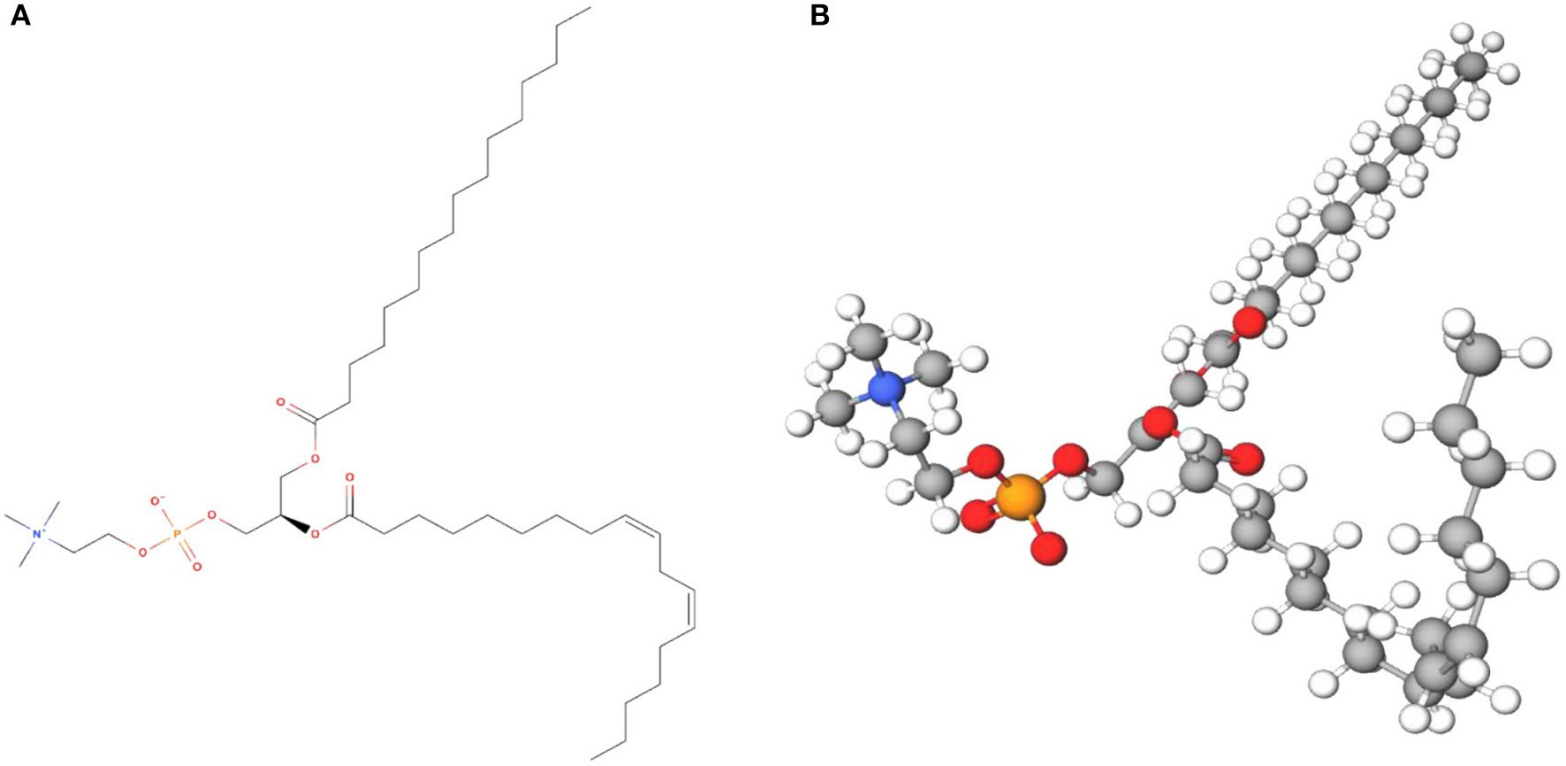
Soybean lecithin (C_42_H_80_NO_8_P) in **(A)** 2D chemical structure and **(B)** 3D model (MolView).

Lecithin can be isolated and characterized from various sources, both from the terrestrial and aquatic (Table 1). These including soybean (48), sunflower (29), rapeseed (26), corn (34), camelina seed (32), canola (31), rice bran (35), egg yolk (37), dairy products (38, 39), and marine products (41, 42) that displaying assorted profile of phospholipids due to variations of two ester-bonded fatty acids at the *sn*-1 and *sn*-2 positions of the glycerol backbone. The *sn*-1 position chiefly carries a saturated fatty acid such as stearic acid or palmitic acid, whereas the *sn*-2 position carries an unsaturated fatty acid such as oleic acid, linoleic acid, α-linolenic acid, arachidonic acid, eicosapentaenoic acid (EPA) and docosahexaenoic acid (DHA) (49). Lecithin from different sources differs substantially on structural and functional roles depending on its origin, either plant or animal origin, and the extraction process (27, 50). An example of chemical structure and model of soybean lecithin is presented in Figure 1.

**Table 1:** Phospholipid content of various lecithin sources.

**Lecithin source**	**Phospholipid content (%)**	**References**
Soybean	45.8–81.9	(26–28)
Sunflower	42.0–64.4	(28–30)
Rapeseed	44.6–77	(26, 28, 31)
Camelina seed	33.8–55.7	(32)
Peony seed	67	(33)
Corn	96	(34)
Rice bran	42.5	(6, 35, 36)
Egg yolk	10–54.3	(27, 37)
Milk and dairy products	0.2–48.4	(38–40)
Krill	30–80	(24, 41)
Mackerel	68.6–84.1	(42)
Anchovy	65	(43)
Salmon	61.1	(26, 44)
Fish roe	64.8–77.9	(27, 45, 46)
Fish by-product	68.9–79.8	(27, 45, 46)
Squid viscera	91.6	(47)
Fish meal	40	(41)

Lecithin is commonly extracted by degumming crude vegetable oils and drying the hydrated gums. Soybean oil, for example, contains approximately 60% acetone-insoluble which corresponds to 2–3% of the commercial lecithin and high amounts of phosphorus (12). Compared to sunflower and rapeseed oils, crude soybean oil has the greatest phospholipid concentration that is still present in vegetable oils after extraction (51). Therefore, soybean oil is the main source for the production of commercial lecithin. A typical crude soybean lecithin is reported to contain 18% PC, 14% PE, 9% PI, 5% phosphatidic acid, 2% minor phospholipids, 11% glycolipids, 5% complex sugars and 37% neutral oil (20). However, these properties are subjected to alteration by deoiling the crude lecithin into high-purity refined lecithin products, or by chemical and enzymatic modification of the phospholipids (52). For examples, the refined soybean lecithin has a greater purity of phospholipid (97.6%) than those from the crude soybean lecithin (60%) (53). Similarly, corn lecithin deoiled by supercritical carbon dioxide is claimed to contain 96% of phospholipids (34).

Animal lecithin derived from eggs, milk and bovine brain contains sphingomyelin on top of the major phospholipid classes such as PC, PE, PS and PI. Egg yolk lecithin is distinguished from soybean lecithin by having relatively higher proportion of saturated fatty acids, *n*-6 and *n*-3 polyunsaturated fatty acids (PUFAs) such as arachidonic acid and DHA. It is even more oxidatively stable than soybean lecithin (54). However, the application of egg yolk lecithin in aquafeed is not a viable option due its cost and commercial availability (37). On the other hand, marine lecithin derived from cold-water fishes is predominated by PC and PE and characterized by the presence of *n*-3 PUFAs such as EPA [20:5(n-3)] and DHA [22:6(n-3)] (55). For instance, marine lecithin extracted from salmon head was found to be rich in phospholipids, especially the PC (44). Nonetheless, supplies of the marine lecithin are not sustainable and therefore much more costly than other sources of lecithin (41, 56). Unlike the animal lecithin, plant lecithin derived from oilseeds such as soybean, rapeseed and sunflower contain mainly of PC, PE and PI and characterized by the availability of mono- and poly- unsaturated fatty acids, namely the oleic acid (18:1), linoleic acid [18:2 (n-6)], and α-linolenic acid [18:3(n-3)] (26). In particular, soybean lecithin has been the most widely used phospholipid source in aquaculture feed formulation due to its market availability and the beneficial effects of promoting growth performance and enhancing survival on fish (57).

## 3. Mode of action

Soybean lecithin is an essential component of aquafeed that exhibits multi-faceted mode of action and involves diverse mechanisms. Primarily used in aquafeed as an emulsifying agent, phospholipid components of soybean lecithin form a protective layer surrounding the lipid droplets in the feed, thus improves the stability of aquafeed and reduces the leaching rate of water-soluble vitamins and minerals (58, 59). By increasing the surface area of the lipid droplets in the aquafeed, soybean lecithin serves to increase the digestibility of aquafeed by making it more easily accessed by digestive enzymes to break down the lipids into smaller molecules. This is particularly important in carnivorous fish species that require a high level of dietary lipids for optimal growth (60). Phospholipids are important for the maintenance of bio-membrane structure, formation of cell organelles and superior to neutral lipids as the main energy source during early life history stages of some fish species (8, 18, 61). Phospholipid deficiency in fish larval diets has been reported to develop aberrant lipid deposition in intestinal enterocytes due to insufficient chylomicron synthesis and reduced specific activity of lipase (62, 63). Besides, phospholipids are reported to suppress cholesterol absorption in the animal digestive system (64) while facilitate the transport of lipids released from the hepatopancreas into hemolymph and enhance the availability of dietary cholesterol in the prawn *Penaeus japonicus* (65).

To ensure that a nutritionally complete and balanced diet is delivered, the mechanism of metabolism for soybean lecithin in aquafeed primarily involves the breakdown of its phospholipid components by digestive enzymes, followed by the absorption of fatty acids and nutrients, and the conversion of choline to betaine. Based on the chemical structure of soybean lecithin, free fatty acids are readily to be absorbed by the fish gut and transported to other tissues for storage, energy production, or used as precursors for various metabolic pathways upon the hydrolysis of ester bonds that link the fatty acid chains to the glycerol backbone of phospholipid (8, 66). Unsaturated fatty acids of soybean lecithin, particularly the omega-3 and omega-6 fatty acids, are crucial for fish growth and health (55). However, choline which typically forms the polar head group of soybean lecithin, is metabolized differently depending on fish species and its nutritional requirements. The conversion of choline to betaine is one of the common pathways that facilitate osmoregulation in fish and profoundly affects a series of metabolic processes including DNA methylation, protein synthesis, lipid metabolism, and energy production (67, 68).

Betaine, the oxidized form of choline, usually serves as a methyl group donor that transfers methyl groups (CH_3_) to metabolic compounds such as DNA, RNA, protein and lipid (69). With the addition of methyl group from betaine and alteration to the structure of DNA molecule, betaine regulates gene expression for growth development and immune responses (70). Furthermore, methylation process is essential for the synthesis of structural proteins and enzymes in aquaculture species. Lacking methyl groups in an organism likely to trigger a condition of under–methylation of DNA and concomitant activation of oncogenes (58, 71). Yet, these methyl groups cannot be synthesized by animals and can only be derived from diet (72). By regulating the activity of enzymes involved in the synthesis and breakdown of fatty acids, betaine impacts on fish lipid metabolism, which modifies the composition of body fat and cell membrane consequently (73). On top of that, betaine is also known to serve as an osmoprotectant in the energy production in fish by lowering the energy demands to regulate ions and cell volume, and supporting the conversion of homocysteine into methionine, an essential amino acid required for protein synthesis (70). Hence, the availability of methyl groups in the diet and functionality of soybean lecithin imparts a number of advantages to aquaculture species.

In addition to its role as the source of fatty acids and choline, soybean lecithin also delivers other important nutrients, such as phosphorus and vitamin E. Phosphorus is an essential mineral that is required for bone formation and other metabolic processes (74), while vitamin E is a powerful antioxidant that can protect cell membranes from oxidative damage (75). By providing a balanced and nutritionally complete diet, soybean lecithin is well-documented to enhance growth performance, survival, proper bone skeletal formation and stress mitigation (76, 77).

Overall, mode of action of soybean lecithin in aquafeed highly relies on its emulsifying properties, as well as its abilities to enhance digestibility and nutritional quality of the aquafeed. Depending on the composition of phospholipid which includes the fatty acids chains, glycerol backbone, phosphate group, and the polar head group, soybean lecithin contributes crucial roles to the growth performance, lipid and carbohydrate metabolisms, nutrient utilization, antioxidant activities and stress resistance in farmed species (78).

## 4. Effects of soybean lecithin on the feed utilization and growth performance of aquaculture species

Extensive studies have been conducted to evaluate the effect of dietary phospholipid in different aquaculture species at different stages by using egg yolk, soybean and marine lecithin in the aquafeed formulation. Of major interest in aquafeed supplementation are the PC, PE and PI (79, 80). Soybean lecithin has been receiving considerable attention in aquafeed formulation due to their implication in numerous metabolic pathways and regulation processes that improve the feed utilization and growth performance of larval and juvenile fish species such as red sea bream, *Pagrus major* (81), knifejaw, *Oplegnathus fasciatus* (81), ayu, *Plecoglossus altivelis* (82), rainbow trout, *Oncorhynchus mykiss* (83), Atlantic salmon, *Salmo salar* (84–86), red drum, *Sciaenops ocellatus* (87), goldfish, *Carassius auratus* (88), common carp, *Cyprinus carpio* (89), seabass, *Dicentrarchus labrax* (74), Japanese flounder, *Paralichthys olivaceus* (90), cobia, *Rachycentron canadum* (91), amberjack *Seriola dumerili*(92), rohu, *Labeo rohita* (68), gilthead seabream, *Sparus aurata* (93, 94), silvery-black porgy, *Sparidentex hasta* (95), large yellow croaker, *Lamichthys crocea* (57), Nile tilapia, *Oreochromis niloticus* (96), hybrid grouper, *Epinephelus fuscoguttatus* x *E. lancolatus* (60), and giant grouper, *E. lanceolatus* (97) (Supplementary Table 1). There are numerous explanations on how the inclusion of soybean lecithin possibly promotes feed utilization and growth performance of the larval aquaculture species. Dietary soybean lecithin, notably phospholipids, has been showing stimulating effects on larval growth and survival when *de novo* synthesis of phospholipids in larval fish was insufficient to meet the requirements of the fish during their early development (98, 99). High phospholipids content in the dietary soybean lecithin not only serves as a superior energy source to the larvae (8), but also promotes the conversion of phospholipids to other lipids, including diacylglycerol, diphosphatidylglycerol, PUFAs, and cholesterol (100). Comparatively, larval stages are more vulnerable to dietary phospholipid deficiency and require more dietary phospholipids than the juveniles. Phospholipids requirements for larval fish varied from 2 to 12% with higher requirements among the marine larval fish (8, 100). As larval stages are highly sensitive to phospholipid deficiency, inclusion of soybean lecithin in microparticulate diet serves an essential role to satisfy phospholipid requirement for ontogenetic development at the expense of fish oil, fish meal and live food. For instance, linoleic acid, a polyunsaturated fatty acid that fish require but cannot be produced on their own, is provided by soybean lecithin (101). Soybean lecithin is known to be easily digested by fish into lysophosphatidylcholine form and absorbed directly by fish (102). The distribution and digestion of ingested free oleic acid (18:1n-9) in fish larvae were significantly influenced by dietary phosphatidylcholine (103, 104). Furthermore, supplemental emulsifier such as soybean lecithin can stimulate the production of lipoprotein in fish digestive system to enhance feed digestibility (105). High PC composition in soybean lecithin functions as an age-dependent feed attractant in enhancing feeding activity to fish larvae and therefore displaying a notable impact on fish development (106, 107).

Besides finfish species, soybean lecithin also demonstrated significant role in supporting good growth performance in a number of larval and juvenile penaeid species, including *Penaeus japonicus* (65, 108), *Penaeus merguiensis* (109)*, Penaeus monodon* (66, 110), *Litopenaeus vannamei* (53, 111–113), and *Macrobrachium rosenbergii* (114, 115). The dietary phospholipid is essential for promoting greater rates of cholesterol turnover from the gut to the circulatory system in crustaceans (116). In recent studies, larval mud crab, *Scylla serrata* (117), juvenile swimming crab, *Portunus trituberculatus* (118, 119) and Chinese mitten crab, *Eriocheir sinensis* (120, 121) also benefited from the inclusion of soybean lecithin that enhances feed utilization, improves survival rate, promotes growth performance and molting frequency. Besides growth performance, positive effect of dietary soybean lecithin is gaining attention on gonadal development of the brood stock such as Chinese mitten crab, *Eriocheir sinensis* (122), swimming crab, *Portunus trituberculatus* (123, 124), red claw crayfish, *Cherax quadricarinatus* (125), and adult sea urchin, *Strongylocentrotus intermedius* (126). Nevertheless, juvenile sea urchin (127) is observed to have reduced weight gain when increased dietary phospholipid levels are provided and these excessive phospholipids eventually converted to neutral lipid in the gut and gonad (Supplementary Table 1).

In feed formulations for juvenile black seabream, *Acanthopagrus schlegeli*, soybean oil may be used up to 60 to 80% in place of fish oil. However, fully replacement of fish oil with soybean oil in the feed formulation is not recommended as it can reduce growth performance in juvenile black seabream (128). This was supported by the studies of Seiliez et al. (129) that growth performance of larval gilthead seabream, *Sparus aurata* was affected when soybean lecithin was utilized as a total replacement of live feed whereas overdose of soybean lecithin in the feed formulation (> 35.6 g/ kg diet) lead to decline in growth performance of early juvenile milkfish, *Chanos chanos* (130). On the other hand, some aquaculture species were found to perform better when marine lecithin was administered instead of soybean lecithin (93, 131). Similar finding was also observed in the study of Salini et al. (132) where juvenile barramundi, *Lates calcarifer* received marine lecithin from krill showed better growth performance than those received soybean lecithin. This may be attributed to the presence of high concentration of PUFAs mainly the EPA and DHA in marine lecithin which are important in promoting growth performance of aquatic animals in the early stage of life (27). As demonstrated by Liu et al. (133), EPA is needed to improve larval growth and survival when DHA level is high but arachidonic acid (ARA) level is low. Although marine lecithin tends to outperform as growth promoter for aquaculture species, soybean lecithin is favored as an alternative growth promoter because it is more viable and economically wise compared to marine lecithin from krill and fish meal. Moreover, study by Jaxion-Harm (56) established that phospholipids derived from soybean lecithin are not significantly different from marine sources such as krill and fish meal in promoting growth performance of Atlantic salmon, *Salmo salar* fry (Supplementary Table 1).

## 5. Effects of soybean lecithin on the abiotic stressors of aquaculture species

Global warming and climate change are issues being highlighted in recent years. These issues have an impact on world aquaculture production in which increased water temperature is one of the abiotic stressors to aquatic animals (134). Sensitivity of fish to xenobiotics is also indirectly influenced by the fluctuations of temperature. Presence of organochlorine pesticides such as endosulfan in the water was found to aggravate the situation by causing a 2.6 to 6°C reduction in thermal tolerance of freshwater fishes (135). Pesticides can seep into the aquatic environment as a result of extensive usage in agricultural activities and it is highly toxic to aquatic animals (136, 137). Thus, the nutritional approach is employed as one of the strategies to enhance the immune system and allow aquatic animals to be resistant to different stressors, especially to thermal stress.

It has been observed that inclusion of dietary soybean lecithin has a beneficial effect on thermal tolerance in aquaculture species which allows the aquaculture species to be more resilient to the fluctuation of water temperature. For instance, Kumar et al. (58) postulated that 1.5 to 2% of soybean lecithin helps milkfish, *Chanos chanos* coping stress from high temperature up to 46.4°C via protection of antioxidative status and neurotransmitter enzymes. Besides, soybean lecithin was observed to allow fish being resistant to stress caused by low temperature (138). Thermal tolerance of aquatic animals is influenced by many factors such as the presence of toxic in the water (139), species of aquatic animals (140), size of aquatic animals (141) and acclimation temperature (142). In this case, antioxidant defense system of fish may be fortified by soybean lecithin and thereby less susceptible to fluctuations in water temperature. Not only serves as the raw material in the repair of cell damage caused by thermal stress, soybean lecithin also promotes the expression of heat shock protein, which offers protection to the cells against the accumulation of altered proteins caused by high or low temperature stress (143–145).

Other than thermal stress, soybean lecithin is found to be effective in helping aquaculture species to cope with stress caused by hypoxic condition (78, 100), changes in water salinity (57), and the presence of pesticide in the water (68). Therefore, supplementation of lecithin from soybean in the feed formulation for aquaculture species is noteworthy to enhance stress tolerance of aquaculture species toward abiotic stressors (Supplementary Table 1).

## 6. Effects of soybean lecithin on the intestinal health, whole body total lipid content, antioxidant capacity and immunity of aquaculture species

Despite those phospholipid and fatty acid components of soybean lecithin are playing important roles in the health maintenance of various aquaculture species, documentation on the effects of soybean lecithin on intestinal health, whole body total lipid content, antioxidant capacity and immunity of aquaculture species are relatively limited in the past. Supplementation of soybean lecithin in the micro diet has been observed to prevent intestinal steatosis in the larval common carp, *Cyprinus carpio* (146), and promotes intestinal health of juvenile red drum, *Sciaenops ocellatus* (87), larval largemouth bass, *Micropterus salmoides* (147), yellow drum, *Nibea albiflora* (148), and adult Nile tilapia, *Oreochromis niloticus* (105). In addition, phospholipid in the soybean lecithin helps to regulate lipid metabolism and increases body lipid content of juvenile amberjack, *Seriola dumerili* (92), large yellow croaker, *Larmichthys crocea* (63), silvery-black porgy, *Sparidentex hasta* (95), hybrid grouper, *Epinephelus fuscoguttatus* × *E. lancolatus* (149). Higher body lipid content is obtained when fatty acids delivery and uptake in the fish are improved (Supplementary Table 1).

In many organisms, antioxidant system functions to mitigate the effects of reactive oxygen species (ROS) by protecting and repairing cells from oxidative damage. Dietary supplementation of soybean lecithin is capable to trigger antioxidant responses in aquaculture species when dealing with oxidative stress, or the elevation of ROS at intracellular level (150, 151). Activities of radical scavenging enzymes such as the superoxide dismutases (SOD), catalase (CAT), glutathione peroxidase (GPX), glutathione-S-transferase (GST) and glutathione reductase (GR) have been used as effective biomarkers to examine the effects of dietary phospholipid on enzymatic function and biochemical pathways in several studies involving larval Dojo loach, *Misgurnus anguillicaudatus* (152), common carp, *Cyprinus carpio* (153), golden mahseer, *Tor putitora* (138), stellate sturgeon, *Acipenser stellatus* (75), gilthead seabream, *Sparus aurata* (94), and hybrid snakehead, *Channa argus x C. maculata* (154). As a consequence of antioxidant capacity-promoting effect of dietary phospholipid, lipid peroxidation is reduced and survival rate is increased in the fish (63).

Dietary supplementation of soybean lecithin is also associated with the enhancement of fish systemic immunity against bacterial infection. According to Adel et al. (153), better immunostimulatory effect was reflected by common carp, *Cyprinus carpio* that received 3% soybean lecithin-enriched diet with an increase of mucosal immune parameters including alkaline phosphatase, lysozyme, protease, and esterase activity. These enzymes are involved in the regulatory secretion of antimicrobial peptides to suppress bacterial pathogens such as *Aeromonas hydrophila, Streptococcus iniae, Yersinia ruckeri*, and *Lactococcus garviea*. The enhancement on immunity and phagocytic activity is further supported by Jafari et al. (155) with an inclusion of 3.3% soybean lecithin for juvenile stellate sturgeon, *Acipenser stellatus*, 6–9% of soybean lecithin for pre-spawning Caspian brown trout, *Salmo trutta caspius* (156), and 0.3 g of soybean lecithin-containing bioemulsifier Lysomax^®^ per kg of diet for the adult Nile tilapia, *Oreochromis niloticus* (105). In contrast, there is almost no observable impact on immune genes expression in golden mahseer fry when diet is supplemented with soybean lecithin (138). Similarly, diet supplemented with 4% soybean lecithin showed limited effects on the innate immune system and whole-body composition of juvenile channel catfish, *Ictalurus punctatus*, despite an improvement on feed conversion (157). Further studies need to be carried on different aquaculture species in order to be conclusive on the effect of dietary soybean lecithin on immunity enhancement.

## 7. Conclusion and future perspectives

Lecithin is obtainable from a wide variety of sources, including terrestrial and aquatic animals and plants. Different sources of lecithin have different compositions of phospholipids and fatty acids which determine the structural and functional roles of lecithin. Despite the fact that marine lecithin contains a high concentration of PUFAs, especially EPA and DHA, soybean lecithin is leading the role as growth promoter in the production of major aquaculture species because it is more practical and cost-effective for aquafeed formulation. Many studies have been included in this review to evaluate the potential of soybean lecithin supplementation and substitution for marine lecithin and live feed in the micro diet of aquaculture species in their early life stages. As concluded from the literature, proper supplementation of phospholipids from soybean lecithin in the larval and juvenile diet have significantly improves growth, survival, feed utilization, lipid metabolism, skeletal formation, stress tolerance, antioxidant capacity, and immune response in a number of finfish and shellfish species from larval to juvenile stage. However, research on the effect of dietary soybean lecithin on pre-reproductive phase and brood stock of crustacean species are relatively lacking. In this regard, future work that address the application of soybean lecithin in the diet formulation for pre-reproductive and brood stock of crustacean species is strongly recommended, with emphasis on enhancing lipid utilization, gonadal development and seed quality.

## Author contributions

Writing—original draft preparation: WW, ZA, and LW. Writing—review and editing: RC, MK, KM, NR, SM, and MR. Supervision: GT-I and LW. All authors contributed to the article and approved the submitted version.
