# Supplementary material for: The roles of soybean lecithin in aquafeed: a crucial need and update
Source: Front Vet Sci. 2023 Sep 15;10:1188659. doi: 10.3389/fvets.2023.1188659 (PMC10546944; doi:10.3389/fvets.2023.1188659)
Supplement: Supplementary file 1 [file Data_Sheet_1.pdf]

Supplementary Table 1. The effects of dietary soybean lecithin on aquaculture species.

Aquaculture species	Initial size/ Life stage	Dose (kg^{-1} diet)	Duration	Findings	References
Red sea bream, *Chrysophrys major*	10 dph larvae	5%	20 days	+ growth performance<br>+ survival rate	(81)
Prawn, *Penaeus japonicus*	Zoea 1 larvae	3– 6%	8 days	+ growth performance<br>+ survival rate	(108, 65)
Prawn, *Penaeus japonicus*	1 g juvenile	3%	30 days	+ growth performance<br>+ lipid utilization	(116)
Giant freshwater prawn, *Macrobrachium rosenbergii*	121 mg juvenile	5%	40 days	± growth performance<br>± survival rate<br>± molting frequency	(114)
Rainbow trout, *Oncorhynchus mykiss*	0.12 g fry	4%	16-20 weeks	+ growth performance<br>+ survival rate<br>+ FCR	(83)
Atlantic salmon, *Salmo salar*	0.18-1.0 g fry	4 – 8%	12-16 weeks	+ growth performance<br>+ survival rate<br>+ FCR	(84)
Atlantic salmon, *Salmo salar*	0.18 g fry	4 – 6 %	14 weeks	+ growth performance	(85)
Pacific white shrimp, *Litopenaeus vannamei*	0.3 mg post larvae	6.5%	18-23 days	+ growth performance	(111)
Red drum, *Sciaenops ocellatus*	2.6 g juvenile	4%	6 weeks	+ growth performance<br>+ feed efficiency<br>+ hepatic lipid	(87)
Goldfish, *Carassius auratus*	0.3 g juvenile	4%	6 weeks	+ growth performance	(88)
Common carp, *Cyprinus carpio*	3 dph larvae	2%	25 days	+ growth performance	(89)
Banana shrimp, *Penaeus merguiensis*	0.1 g juvenile	1 – 2%	8 weeks	+ growth performance<br>+ survival rate<br>+ feed efficiency	(109)

Black tiger prawn, *Penaeus monodon*	Zoeal, mysid larvae, 15-day-old postlarvae	1 – 1.5%	Until PL-15	+ growth performance<br>+ survival rate<br>+ tolerance to low salinity	(66)
Pacific white shrimp, *Litopenaeus vannamei*	750 mg juvenile	3 – 5%	6 weeks	+ growth performance<br>+ total lipid in hepatopancreas<br>– total lipid in muscle	(53)
Sea bass, *Dicentrarchus labrax*	9 dph larvae	56 – 167 g	40 days	+ growth performance<br>+ survival rate<br>– skeletal deformity	(74)
Giant freshwater prawn, *Macrobrachium rosenbergii*	2 mm larvae	1.5%	28 days	± body length<br>+ survival rate	(115)
Black tiger shrimp, *Penaeus monodon*	1.34 g juvenile	20 g	42 days	+ growth performance<br>+ lipid digestibility<br>± survival rate	(110)
Gilthead seabream, *Sparus aurata*	4 dph larvae	90 – 160 g	20 days	± survival rate<br>– growth performance<br>+ abnormal liver and calculi in the urinary bladder	(129)
Japanese flounder, *Paralichthys olivaceus*	1 g juvenile	20 – 40 g	40 days	+ growth performance	(90)
Mud crab, *Scylla serrata*	Megalopa	40 g	10 days	+ growth performance<br>+ survival rate<br>– development time	(117)
Cobia, *Rachycentron canadum*	0.4 g larvae	8%	42 days	+ growth performance<br>+ survival rate<br>+ lipoprotein lipase and hepatic lipase activities	(91)
Pikeperch, *Sander lucioperca*	10 dph larvae	13%	24 days	+ growth performance<br>+ digestive tract maturation<br>± skeletal deformity	(101)
Amberjack, *Seriola dumerili*	2.6 g juvenile	20 – 40 g	30 days	+ growth performance<br>+ feed intake	(92)

Chinese mitten crab, *Eriocheir sinensis*	110 g broodstock	2.4 – 3.6%	7 months	+ ovarian development<br>+ reproductive performance	(122)
Sea urchin, *Lytechinus variegatus*	0.09 g juvenile	8.8%	12 weeks	– growth performance<br>+ neutral lipid deposition in the gut and gonad	(127)
Gilthead seabream, *Sparus aurata*	32 dph larvae	5%	1 week	– growth performance<br>+ lipolytic activity	(131)
Pacific white shrimp, *Litopenaeus vannamei*	0.08 g juvenile	3%	10 weeks	+ survival rate	(112)
Rohu, *Labeo rohita*	8 g fingerling	2%	5 weeks	+ growth performance<br>– stress induced by endosulfan toxicity	(68)
Rainbow trout, *Oncorhynchus mykiss*	120 mg fry	40 g	40 days	+ growth performance<br>+ survival rate<br>+ digestive enzyme activity	(79)
Redclaw crayfish, *Cherax quadricarinatus*	25.6 g broodstock	2 – 6%	8 weeks	+ ovarian development	(125)
Large yellow croaker, *Lamichthys crocea*	15 dph, 3 mg larvae	50 – 100 g	30 days	+ growth performance<br>+ survival rate<br>+ digestive enzyme activity<br>– stress induced by declining water temperature and salinity fluctuation	(57)
Milkfish, *Chanos chanos*	13 g fingerling	1 – 2%	5 weeks	+ thermal tolerance and protection against cellular stress induced by endosulfan	(58)
Dojo loach, *Misgurnus anguillicaudatus*	15 dph, 10 mg larvae	6 – 8%	30 days	+ growth performance<br>+ survival rate<br>+ antioxidant response	
Channel catfish, *Ictalurus punctatus*	5.8 g juvenile	2 – 4 %	42 days	± growth performance<br>± innate immune response	(157)

Swimming crab, *Portunus trituberculatus*	22 g juvenile	1 – 4%	8 weeks	+ growth performance<br>± survival rate<br>± FCR<br>± molting frequency	(118)
Blunt snout bream, *Megalobrama amblycephala*	0.35 g fingerling	6%	60 days	+ growth performance<br>± survival rate<br>± FCR<br>± antioxidant response	(59)
Gilthead seabream, *Sparus aurata*	16 dph larvae	90 g	29 days	+ growth performance<br>+ survival rate<br>+ peroxidation risk<br>+ skeletal deformities	(93)
Atlantic salmon, *Salmo salar*	First – feeding fry (900 °day post – fertilization)	3.2 – 3.6%	From first feeding fry to parr-smolt transformation	+ growth performance in fish up to 2.5 g;<br>± growth performance in fish above 2.5 g to smolt<br>– vertebral deformity	(80, 86)
Barramundi, *Lates calcarifer*	47 g juvenile	72 g	56 days	± growth performance<br>– FCR<br>– lipid digestibility<br>+ expression of genes related to fatty acid metabolism	(132)
Large yellow croaker, *Larimichthys crocea*	3.86 mg larvae	5 – 12%	30 days	+ antioxidant capacity<br>+ regulate lipid metabolism	(63)
Swimming crab, *Portunus trituberculatus*	25.4 g juvenile	15 g	50 days	± growth performance<br>+ whole body and serum triglyceride levels<br>+ muscle and hepatopancreas fatty acid profiles	(119)
Swimming crab, *Portunus trituberculatus*	25.5 g broodstock	1 – 8%	16 weeks	+ ovarian development<br>+ expression of *Pt – fabp* and *vtg* genes in hepatopancreas	(123)

Common carp, *Cyprinus carpio*	14 g	30 g	8 weeks	+ growth performance<br>+ survival rate<br>+ digestive enzymes activity<br>+ antioxidant activity<br>+ antibacterial activity<br>+ mucosal immune activity	(153)
Stellate sturgeon, *Acipenser stellatus*	11.3 g juvenile	6%	75 days	+ growth performance<br>± survival rate<br>+ innate immunity	(155)
Milkfish, *Chanos chanos*	9.0 mg juvenile	35.6 g	50 days	+ growth performance<br>+ survival rate	(130)
Hybrid snakehead, *Channa argus* × *C. maculata*	12.6 g juvenile	8.5 – 41.5 g	8 weeks	+ growth performance<br>+ antioxidant capacity<br>– hepatic lipid deposition	(154)
Caspian brown trout, *Salmo trutta caspius*	350 g pre-spawning	6 – 9%	90 days	+ growth performance<br>+ innate immunity<br>+ digestive enzyme activity	(156)
Silvery– black porgy, *Sparidentex hasta*	38 g juvenile	60 – 90 g	56 days	+ growth performance<br>+ survival rate<br>+ whole body total lipid content	(95)
Swimming crab, *Portunus trituberculatus*	25.5 g juvenile	40 – 80 g	16 weeks	+ growth performance<br>+ antioxidant capacity<br>+ lipid metabolic enzyme activity<br>+ vitellogenesis in pre-reproductive phase female	(124)
Chinese mitten crab, *Eriocheir sinensis*	0.26 g juvenile	30 g	8 weeks	+ growth performance<br>+ molting frequency<br>+ antioxidant capacity<br>+ lipid utilization	(120)

Chinese mitten crab, *Eriocheir sinensis*	0.53 g juvenile	10 – 50 g	8 weeks	+ lipid utilization	(121)
Golden mahseer, *Tor putitora*	141 mg fry	2%	70 days	± growth performance<br>± survival rate<br>± immune gene expression<br>+ antioxidant capacity<br>+ thermal tolerance	(138)
Nile tilapia, *Oreochromis niloticus*	70.3 g adult	0.45 g commercial soybean lecithin – containing bioemulsifier, Lysomax®	60 days	+ growth performance<br>+ feed utilization<br>+ intestinal health<br>+ innate immune response	(105)
Stellate sturgeon, *Acipenser stellatus*	11.3 g juvenile	6%	75 days	+ growth performance<br>+ digestive and oxidative stress enzyme activities<br>– hepatic lipid deposition	(75)
Atlantic salmon, *Salmo salar*	0.14 g fry	4.03%	63 days	± growth performance	(56)
Rainbow trout, *Oncorhynchus mykiss*	120 g	2 – 4% (with conjugated linoleic acid)	74 days	+ resistance to hypoxic stress	(78)
Milkfish, *Chanos chanos*	18 dph larvae	52.5 – 70 g	6 weeks	+ growth performance<br>+ survival rate<br>+ intestinal lipase activity<br>+ FCR<br>– opercular malformations	(77)
Hybrid grouper, *Epinephelus fuscoguttatus* × *E. lancolatus*	40 dph, 0.37 g larvae	20 – 80 g	23 days	+ growth performance<br>+ antioxidant capacity<br>+ hepatic lipid deposition	(60)

				± expression of lipogenesis related genes<br>± expression of fatty acids oxidation related genes	
Hybrid grouper, *Epinephelus fuscoguttatus* x *E. lancolatus*	13 g juvenile	20 – 80 g	61 days	± growth performance<br>± feed utilization<br>+ lipid deposition in whole fish body and liver<br>± expression of lipogenesis related genes<br>– expression of fatty acids oxidation related genes	(149)
Giant grouper, *Epinephelus lanceolatus*	68.6 g	10 g	6 weeks	± growth performance<br>+ nutrient digestibility<br>– hepatic lipid deposition	(97)
Gilthead seabream, *Sparus aurata*	0.49 g fingerling	40 g (fortified with ascorbic acid)	60 days	+ growth performance<br>+ feed utilization<br>+ antioxidant capacity	(94)
Yellow drum, *Nibea albiflora*	5.0 mg larvae	12%	28 days	+ growth performance<br>+ mid-intestinal morphology	(148)
Rock bream, *Oplegnathus fasciatus*	15.1 mg larvae	12%	30 days	+ growth performance<br>+ survival rate<br>+ stress resistance to hypoxia<br>+ intestinal morphology<br>+ expression of genes related to fatty-acid activation, phospholipid catabolism, amino acid synthesis, and ribosome biogenesis	(100)
Sea urchin, *Strongylocentrotus intermedius*	21 g, two year–old adult	1.5%	30 days	+ growth performance<br>+ antioxidant capacity	(126)

White shrimp, *Litopenaeus vannamei*	1.02 g juvenile	1.8%	8 weeks	+ gonadal development and gametogenesis in the male; slower in the female<br>+ growth performance<br>+ hemolymph parameters<br>+ body texture<br>+ immune responses	(113)

Note: + indicates increased, or improved response; – indicates decreased, or reduced response; ± indicates no significant differences compared to the control and/ or other treatments; FCR, feed conversion ratio
